# Noninvasive Biomarkers of Gut Barrier Function in Patients Suffering from Diarrhea Predominant-IBS: An Update

**DOI:** 10.1155/2020/2886268

**Published:** 2020-10-13

**Authors:** Michele Linsalata, Giuseppe Riezzo, Caterina Clemente, Benedetta D'Attoma, Francesco Russo

**Affiliations:** Laboratory of Nutritional Pathophysiology, National Institute of Gastroenterology “S. de Bellis” Research Hospital, I-70013 Castellana Grotte, Italy

## Abstract

The intestinal barrier plays a crucial role in the absorption of nutrients and in preventing the entry of pathogenic microorganisms and toxic molecules. Several studies have shown a compromised intestinal barrier associated with low-grade inflammation in the small intestinal mucosa in celiac disease, inflammatory bowel disease, and irritable bowel syndrome (IBS), particularly in IBS with diarrhea (IBS-D). In light of these new data, IBS is no longer considered a functional disease but rather a heterogeneous syndrome that has yet to be carefully studied. Therefore, investigating the integrity and function of the intestinal barrier is now essential to improving knowledge of the pathophysiology of IBS-D and to improving the management of IBS-D patients. However, the study of the intestinal barrier must clarify some still unsolved methodological aspects and propose standardised assays before becoming a useful diagnostic tool. In this framework, this review will discuss data about the tests that noninvasively evaluate the integrity and functionality of the human intestinal barrier, paying particular attention to patients with IBS-D, in both clinical and research situations.

## 1. Introduction

The intestinal epithelium is the main barrier that separates our body from the external environment. Consequently, the structural and biochemical constituents of the intestinal mucosa, often referred to as the “gut barrier,” play a critical role in the absorption of nutrients, electrolytes, and water, and they prevent the entry of pathogenic microorganisms and toxic luminal substances [1]. Intestinal permeability (IP) is a functional feature of the intestinal barrier and may be defined as the ability of the mucosal surface to be penetrated by specific substances. In general, two distinct IP pathways are recognised. The transcellular pathway allows solute transportation across the enterocyte's membrane, and the paracellular pathway is regulated by tight junctions (TJs) [2]. These adhesion complexes between the cells of the intestinal epithelium are composed of the transmembrane proteins occludin and claudins, which interact with zonula occludens proteins. These proteins bind directly to the actin cytoskeleton to control the passage of molecules through the paracellular space [3]. Zonulin is a protein that regulates TJs, and upon stimulation of luminal factors, including food and bacterial toxins, it acts on the apical receptors to increase permeability and facilitate absorption [4].

The intestinal barrier can be compromised through severe structural damage to the mucosa or functional alteration in the regulating components of the barrier. A consequence of perturbations in the gut barrier can lead to increased IP, which may challenge the immune system of susceptible individuals and affect the host-microbial balance, thus inducing inflammatory changes in the target organs. This “leaky gut hypothesis” seems to be a reasonable explanation of the pathophysiological background of various diseases [5]. In this framework, an increasing number of reports have provided strong evidence of a compromised intestinal barrier associated with low-grade inflammation in the upper intestinal mucosa in patients with irritable bowel syndrome (IBS). This syndrome is a functional disorder of the gastrointestinal (GI) tract and is characterised by abdominal pain and alterations in bowel habits [6, 7]. Notably, the data in the literature show that in the IBS with diarrhea (IBS-D) group, which accounts for about one-third of all IBS cases [8], IP is more pronounced and is associated with visceral hypersensitivity, suggesting breakdown of the epithelial barrier as an early event [9, 10]. Insight into the loss of gut barrier integrity and function is imperative for expanding our knowledge on disease aetiology and pathophysiology, as well as for improving the clinical management of IBS-D patients. An invasive procedure, such as the collection of a biopsy, is inconvenient to the patient and usually implies high healthcare costs.

Conversely, many analytes could be used to noninvasively investigate gut barrier function, and they could be particularly attractive for patient monitoring (Table 1). However, these tests are not yet used in routine clinical chemistry [11–13]. The findings regarding an altered intestinal barrier in IBS patients are different among studies since not only the patients enrolled in the clinical trials have different clinical profiles but also the evaluation of the intestinal integrity and permeability was made using different methods [14]. In addition, efforts to standardise permeability tests for the entire intestine continue to be made to obtain a reliable test. In this framework, this review will discuss data about the tools that noninvasively evaluate the integrity and function of the human intestinal barrier, paying particular attention to patients with IBS-D, in both clinical and research situations.

## 2. Biomarkers of Epithelial Cell Damage

### 2.1. Intestinal Fatty Acid-Binding Protein

A lining of enterocytes and TJs maintains the intestinal barrier; therefore, loss of intestinal barrier integrity can be assessed by different tests that investigate epithelial cell damage. Intestinal fatty acid-binding protein (I-FABP) is a 15 kDa cytosolic protein that plays a role in the cellular uptake and metabolism of fatty acids. It is located in mature enterocytes on the tip of the intestinal villi, which is the anatomical region that is first affected by mucosal damage [15, 16]. Under physiological conditions, the circulating levels of I-FABP are low, and it is eliminated through urine. It is released after mucosal tissue injury, as soon as cell membrane integrity is compromised, thus representing a marker of impaired transcellular permeability. It subsequently appears in the circulation, making it a potential candidate as a very early diagnosis marker [17, 18]. Many studies have suggested that I-FABP in serum or urine might be a useful biochemical marker for the diagnosis of ischaemic intestinal injury in humans, and a relationship between blood I-FABP concentration and GI diseases has been reported [19–22]. In particular, in celiac disease (CD), I-FABP could be considered a noninvasive marker for the detection of architectural mucosal anomalies and could provide evidence that these abnormalities contribute to increased IP [23, 24]. Furthermore, I-FABP seems to reflect the early gluten-induced damage, and I-FABP evaluation could be useful to monitor disease activity during follow-up [25, 26]. Increased circulating I-FABP levels were also found in individuals with nonceliac wheat sensitivity (NCWS), indicating increased intestinal epithelial cell damage and increased microbial translocation in such patients [27].

Few data exist in the literature regarding I-FABP and IBS. Recently, patients with postinfectious IBS (PI-IBS) showed significantly higher I-FABP levels than non-postinfectious IBS (NPI-IBS) patients and healthy controls (HCs). Moreover, the I-FABP levels found in the subgroup of PI-IBS patients with diarrhea were significantly higher than the I-FABP levels of the NPI-IBS subgroup [28]. In a recent study by our group [29], serum levels of I-FABP were evaluated in duplicate by enzyme-linked immunosorbent assay (ELISA) (Thermo Fisher Scientific, Waltham, MA, USA) (intra-assay coefficient of variation < 10%; interassay coefficient of variation < 12%; standard: 25–0.024 ng/mL; sensitivity: 25 pg/mL). A significant positive correlation between I-FABP and variations in small IP, expressed by the lactulose/mannitol ratio, was found. Moreover, IBS-D patients and HC subjects had significantly lower serum levels of I-FABP than patients with CD.

Interestingly, the IBS-D patients with altered small IP showed similar values of I-FABP as the CD patients and significantly higher I-FABP values than the IBS-D patients with normal small IP and the HCs. This evidence suggests the loss of integrity of the intestinal epithelium in the IBS-D patients with altered small IP and, consequently, the existence of two distinct IBS-D subtypes. The blood evaluation of I-FABP could provide information on the state of health of the intestinal epithelium, allowing multiple measurements over time and with a definite advantage for the patients in terms of follow-up.

### 2.2. Diamine Oxidase

Diamine oxidase (DAO) is an amine oxidase that catalyses the oxidative deamination of primary amines to form the corresponding aldehydes, ammonia, and hydrogen peroxide. This enzyme is located in villus tip enterocytes of mammals, and its activity increases successively from the duodenum to the ileum [30]. DAO acts as a plasma marker of small bowel mucosa integrity and impaired transcellular permeability in different pathological conditions [31–33]. In addition, this enzyme may be useful to detect and quantify small bowel mucosal atrophy in patients with malabsorption syndromes [34], CD [35–37], and inflammatory bowel disease (IBD) [38, 39].

A cross-sectional study of IBS showed that serum DAO levels in IBS-D were significantly higher than those in the controls [40]. These results were not confirmed in a recent case-control study by our group [29] in which serum DAO levels were not significantly different between IBS-D patients and HC. Conversely, when we categorised the patients according to altered or normal small IP, IBS-D patients with altered small IP showed significantly higher DAO levels than IBS-D patients with normal small IP. These results suggest, once again, that IBS-D patients do not represent a homogeneous group in terms of small intestinal integrity. Given the limited experience in the clinical use of this barrier marker, it may be difficult to compare these results with previous studies; therefore, serum DAO levels need to be verified by further investigations in IBS patients.

### 2.3. TJ Proteins

Loss of gut wall integrity, including the breakdown of TJs, is an early event in intestinal damage. The normal expression of TJ proteins is crucial for the maintenance of gut barrier function. In contrast, decreased TJ levels or impaired TJ assembly results in barrier dysfunction and increased GI epithelial paracellular permeability. Therefore, markers for the loss of TJ integrity can be helpful in the elucidation of the pathophysiology of diseases in which injury of the gut plays a key role [41, 42].

Clinical and experimental studies have demonstrated that defects in the intestinal TJs can occur in both IBD and IBS [10, 43–45]. MicroRNAs (miRNAs), which are noncoding regulators of gene expression at the posttranscriptional level, play an essential role in targeting transcripts that encode proteins of intestinal TJs and their regulators [46]. In patients with IBS, the enhancement of gut paracellular permeability is also associated with an increase in the levels of several miRNAs [47].

Notably, the upregulation of claudin-2 expression in the ileum may be an important pathophysiological factor related to IBS-D development [48, 49]. Moreover, a strong association between the gene and protein expression of TJs and IP, mast cell biology, and clinical symptoms has been found in IBS-D patients [50, 51]. All these data have been obtained by invasive methods on tissue samples or biopsies. Since TJ protein loss and successive urinary excretion have been observed during epithelial turnover and barrier alteration, it has been hypothesised that noninvasive methods for the detection of TJ breakdown could help in the early diagnosis and follow-up of patients with alterations of the intestinal barrier.

In a pilot study conducted on patients with IBD, elevated urinary claudin-3 levels were observed along with reduced intestinal staining of the protein. Also, a correlation between urinary claudin-3 levels and disease severity was reported [52]. To date, there are no data on this topic in patients with IBS. Therefore, clinical and experimental studies of the urinary levels of TJ proteins in diseases associated with intestinal barrier defects, such as IBS-D, should be encouraged, taking into account that the collection and analysis of serum or faecal samples could also represent a noninvasive strategy to obtain information of the health of the intestinal barrier in IBS-D.

## 3. Functional Tests to Assess Intestinal Permeability

It is known that IP can be assessed with accurate and specific methods, which require an invasive approach. Among them, the Ussing chamber system offers an *ex vivo* measurement of GI epithelium permeability using fluorescent probes as well as electrophysiological measurements [53]. The advantage of Ussing chambers is their ability to study how all mucosal sheets can interact together in modulating the intestinal mucosal barrier. In addition, pharmaceutical blockers and stimulators can be used to determine the role of the individual components in the mucosal tissue. However, the invasive approach and the lack of readily available human tissues, especially from healthy individuals, represent limitations to this technique [54].

On the other hand, the assessment of IP can be carried out *in vivo* through several noninvasive techniques based on the oral administration of various probes and their subsequent variable, timed urinary recovery. Usually, these probes are differentially transported across the intestinal epithelium. They get through the intestinal mucosa by transcellular or paracellular routes, reaching the circulation, and are filtered by the kidney, thus allowing their evaluation in the urine. The time of urine collection reflects the intestinal segment crossed by the probes [11]. There are several types of markers for the assessment of intestinal barrier permeability, including sugars, radioisotopes such as 51-Chromium-labeled Ethylene Diamine Tetra-acetic Acid (^51^Cr-EDTA), and polyethylene glycols (PEG). Another interesting probe for the functional assessment of intestinal barrier loss is zonulin because of its ability to regulate TJs.

### 3.1. Sugar Probes

Today, the most used methods for assessing GI permeability involve the use of small sugar molecules of different sizes, and sucrose (Su), lactulose (La), mannitol (Ma), rhamnose (R), and sucralose (S) are the most used. Su is a disaccharide hydrolysed by the enzyme sucrase in the jejunum, and it can provide information on gastroduodenal permeability. La is another disaccharide that is not broken down by human enzymes, and it can permeate by the paracellular route, while Ma and R are monosaccharides that can permeate by the transcellular route. These sugars are degraded by bacteria that are most abundant in the colon, and their 0–5 h urinary fractions are currently used as a marker for small IP. S, a disaccharide, and erythritol (E), a sugar alcohol, are not broken down by human enzymes nor by human colonic bacteria; their 5–24 h and 0–24 h urine collections are used as indicators for colonic and whole gut permeability, respectively [55, 56].

The La/Ma ratio represents the most used functional measurement of small IP in humans and represents a more specific index for comparisons between groups. The combination of the two sugars can be used to eliminate factors related to uptake, distribution, or excretion, which may obscure the differences between subjects [57]. For the dosage of these sugars in the urine, the most common laboratory methods used are high-performance anion-exchange chromatography with pulsed amperometric detection (HPAEC-PAD) and liquid chromatography-mass spectrometry (LC-MS) [58, 59].

Altered IP was observed in numerous GI and non-GI diseases using different sugar absorption tests [60, 61]. In IBS patients, alterations in IP appear to be subtype-related and site-specific. In particular, IBS-D patients seem to be characterised by an increase in small IP compared to HCs. However, these results are not always consistent, and *in vivo* data on colonic permeability are contrasting since lower, higher, or unaltered permeability was found when comparing IBS-D to HCs [62–64].

A validated multisugar test was recently used to simultaneously measure IP at various sites in the GI tract [65]. This test allows the administration of five different sugars as indicators of gastroduodenal permeability, the small intestine, the colon, and the entire intestine. The subsequent analysis is carried out in two urine fractions, namely, 0–5 h and 5–24 h. Significantly higher urinary Su levels were found in IBS-Total, IBS-D, and IBS with constipation (IBS-C) patients compared to controls. In addition, significantly higher La/R ratios have been found in IBS-D patients compared to controls. After adjustment for possible confounders (body mass index, smoking history, medication, and sex), only the La/R ratio, representing small IP, increased when compared to IBS-D and controls. No difference was found in the 5–24 h and 0–24 S/E ratio between groups. This finding is in line with previous observations in studies with small sample sizes [64] and points to a different pathophysiological mechanism in IBS-D compared to other IBS subtypes.

The increase in the permeability of the small intestine seems to also be pivotal for the gluten intolerance described by some IBS patients. It is plausible that the link between gluten or gliadin and inflammation may cause the increase in IP. A previous study also suggested an immunogenetic predisposition to gluten intolerance in a subgroup of patients with IBS-D in the absence of CD [66]. Consistent with this hypothesis, a randomised controlled 4-week trial in patients with IBS-D suggested that a gluten-containing diet was associated with higher small bowel permeability (evaluated by La and Ma excretion) with respect to a gluten-free diet (GFD). In addition, small bowel permeability was higher in HLA-DQ2/8-positive patients than in HLA-DQ2/8-negative patients, and no significant differences in colonic permeability were observed. These findings suggest that gluten alters bowel barrier functions in patients with IBS-D, particularly in HLA-DQ2/8-positive patients, and these findings reveal a reversible mechanism for the disorder [67]. Another study of IBS-D patients did not confirm the influence of the HLA type on mucosal permeability (La/Ma), TJs, mRNA expression, or small bowel mucosal morphology [68]. These data are consistent with the hypothesis that gluten sensitivity may represent a separate entity in the spectrum of IBS-D patients.

Recently, IP evaluation of the small bowel by La/Ma identified IBS patients with alterations in gut barrier function. This subgroup is characterised by increased GI permeability and/or production of interleukin- (IL-) 10. These alterations also reflect more severe IBS, as measured by the interference of IBS with daily activities and daily IBS symptoms [69].

In this framework, some recent studies have observed a distinction between different phenotypes with increased or normal small IP in IBS-D patients. In a study by Mujagic et al. [65], small IP remained significantly increased in IBS-D patients compared with controls, even if an overlap between these two groups was observed, which meant that the small IP of the IBS-D patients was partly increased. Zhou et al. [70] reported that approximately 39% of IBS-D patients had increased small IP. A notable finding of this work is that patients with increased membrane permeability also had a higher IBS severity index as well as increased visceral and thermal hypersensitivity to experimental nociceptive pain stimuli. Subsequently, the same authors showed that a subset of IBS-D patients (42%) had increased IP and decreased glutamine synthetase expression compared to that of IBS-D patients with normal permeability and compared to controls [71].

Similarly, another study [72] measuring GI permeability using triple sugar probes found that the proportion of IBS-D patients with increased small IP (47%) compared to the HCs tended to have higher anxiety and depression scores and reduced quality of life compared to patients with normal small IP. A recent work [29] showed significant differences in the biomarker profiles related to the intestinal barrier function between HCs, IBS-D patients, and CD patients. The study also found that 46% of IBS-D patients showed higher small IP, as diagnosed by the La/Ma ratio, with a value higher than the cut-off of 0.035. Interestingly, these patients had significantly higher levels of La (%), Su (%), I-FABP, and DAO compared to IBS-D patients with normal IP, despite the absence of significant differences in the symptom profile between the two subpopulations. The inflammatory parameters and markers of bacterial translocation, namely, IL-6, IL-8, and lipopolysaccharides (LPS), were significantly higher in patients with increased small IP than in normal patients. Russo et al. [73] also observed different levels of adipo(cyto)kines between IBS-D patients with altered IP and IBS-D patients with normal IP, hypothesising that even molecules secreted by adipose tissue could have an impact on the intestinal barrier function.  Table 2 lists previous studies that have identified two subtypes of IBS-D patients using the La/Ma ratio.

The attenuation of the inflammation and preservation of the impaired mucosal barrier function may be an attractive therapy. A randomised, double-blind, placebo-controlled trial found that dietary glutamine supplements dramatically and safely reduced all major IBS-related endpoints (e.g., raw IBS symptom score, stool frequency, and stool form) and normalised the IP (utilising a cut-off value of 0.07 for the La/Ma ratio) in patients with IBS-D with increased IP following intestinal infection [74]. Promising therapy relies on the use of the probiotic bacteria that interact with the host epithelium to resolve inflammation and preserve the barrier function [75]. In a single-blind, randomised, placebo-controlled study, probiotic treatment significantly decreased small bowel permeability, measured by a triple sugar test. Moreover, treatment with probiotics significantly reduced the mean global IBS scores compared with the baseline scores, suggesting that short-term active lactic acid bacteria treatment for IBS-D improved mucosal barrier function [76].

### 3.2. ^51^Cr-EDTA

^51^Cr-EDTA has physiological properties that are similar to oligosaccharides, with the advantage of being easily detectable. Bacteria do not degrade ^51^Cr-EDTA in the colon, which makes ^51^Cr-EDTA a useful marker for both small and large IP. Furthermore, this probe has the advantage of accurate label quantification in urine since it does not require prior extraction from a biological fluid. Unfortunately, ^51^Cr-EDTA relies on radioactivity; therefore, it cannot be utilised for research purposes in children and screening in healthy subjects. In addition, it should be noted that the test involves a single probe to measure paracellular permeability of the intestine rather than a more commonly used double or triple sugar test. Consequently, the recovery of ^51^Cr-EDTA in urine could potentially be affected by nonmucosal factors, such as the gastric emptying rate, intestinal transit time, and renal clearance [77].

Increased urinary recovery of oral ^51^Cr-EDTA has been established in a range of intestinal and non-GI conditions [78, 79]. Nevertheless, there were not significant differences in proximal and distal small IP in IBS-D patients compared to controls. At the same time, the colonic permeability of IBS-D patients was significantly higher than that of HCs and correlated with increased stool frequency, suggesting that a defective intestinal barrier may contribute to the development of GI symptoms [80]. On the contrary, after collecting urine for 24 h, it was observed that proximal small IP increased in IBS-D patients compared to patients with IBS-C and HCs. Moreover, small IP was more likely in NPI-IBS with diarrhea patients compared with PI-IBS patients or HCs, suggesting that those without a history of infectious onset have a more severe defect [81]. The discrepancy in the results between these studies could be due to the extreme variations in the normal range of IP observed in asymptomatic controls using ^51^Cr-EDTA [82]. Thus, further investigation is needed to make this test reliable for investigating IP in IBS-D patients.

### 3.3. PEG

Another commonly used option for IP analysis is based on the use of PEG probes of different sizes [83]. PEG has several advantages over the use of ^51^Cr-EDTA and sugars. It is not radioactive, it is not metabolized by enzymes or degraded by bacteria within the human GI tract, and the required method of analysis is less expensive and time-consuming than those for the other probes. However, the addition of PEG to some food products, such as artificially sweetened sodas, and its occasional use in clinical practice for colonic lavage limits its applicability. PEG has also been criticised for not being sufficiently sensitive to assess IP in some subtle disorders of barrier function due to the low urine excretion of PEG 400 [84]. The molecular components of differing sizes cross the intestinal epithelium at different rates, and PEG probes are suggested to be particularly suitable markers for the whole gut permeability assessment in a broad range of intestinal diseases, although both decreased and increased IP have been found [85–89]. This discrepancy could be due to the use of different sized probes and the transcellular or paracellular permeation pathway studied. Nevertheless, it has been demonstrated that GI permeability tests based on the urinary excretion of different PEG probes and the La/R ratio show equivalent performance in healthy individuals after the consumption of nonsteroidal anti-inflammatory drugs [90]. Interestingly, the PEG test and the La/R test gave equivalent results in CD patients, with abnormal permeability at presentation, normalising during the GFD, and again with altered results during gluten challenge [91].

In regard to IBS, it has been observed that when using both PEG and La/Ma as tests, there are no significant differences in IP between subjects with IBS and HCs [92]. Conversely, by employing the differential urinary excretion of the two PEG molecules (the PEG 3350/PEG 400 ratio) as an “IP index,” Park et al. [93] found that IP increased significantly in patients with IBS compared with HCs. No significant difference in IP was observed among patients with IBS-D, IBS-C, and mixed IBS (IBS-M). The possible causes for this discrepancy are the bias from the small number of patients and the different periods for urine collection, in addition to the different probes used. In light of these data, it is clear that future studies on the permeation pathways of PEG are necessary to improve the interpretation of results, particularly in IBS-D patients.

### 3.4. Zonulin

Considerable knowledge exists about the ultrastructure of TJs, but relatively little is known about their pathophysiological regulation, which leads to local and systemic inflammation [94].

Zonulin is the human protein that is known to regulate the paracellular pathway reversibly by changing the TJ protein-protein interactions. This protein transactivates the epidermal growth factor receptor through proteinase-activating receptor-2, with subsequent TJ disassembly and increased permeability [4].

Some potential intestinal stimuli, such as intestinal bacteria and gluten, can increase zonulin secretion, and this protein plays a particular role in the pathogenesis of numerous diseases. It has been studied as a peripheral marker of IP and inflammation [95–97], particularly in CD [98, 99], where the prophylactic efficacy of zonulin inhibitors, i.e., larazotide acetate, as well as a correlation between zonulin levels and IP has been observed [100, 101]. The role of zonulin in the pathophysiology of IBS has not been well studied, and contradictory results have been provided in this regard. A preliminary study showed that serum zonulin levels found in patients with IBS-D were similar to those observed in HCs [102]. These data were confirmed by a study in which no differences in zonulin levels were found, even between IBS-D patients with normal or altered IP [29].

On the contrary, it has recently been observed that IBS-D patients have similar serum levels of zonulin as CD patients and higher serum levels of zonulin compared to HCs. Although the zonulin levels did not correlate with the overall severity of IBS symptoms, they were positively correlated with stool frequency per week and dissatisfaction with bowel habits [103]. Furthermore, a study by Bueno [104] described IBS-D patients with increased serum zonulin levels and suggested that zonulin signaling via protease-activated receptor 2 may be involved in the pathogenesis of IBS-D.

The discrepancy among the data on serum zonulin levels in patients with IBS-D could be attributable to the presence of more than 50 different proteins participating in the regulation of TJs and IP [105]. For this reason, caution must be taken before considering serum zonulin as a reliable biomarker of IP.

Faecal zonulin may be more associated with IP since secretion of zonulin from the intestinal barrier may leak into the lumen, whereas serum zonulin originates from several different tissues. In fact, in a recent study, a reduction in faecal zonulin was observed in patients with IBS-D after treatment with symbiotics. At the same time, no correlation was found between serum and faecal levels of zonulin [106]. Although several studies have indicated that elevated zonulin levels represent a disease biomarker [107, 108], the degree of correlation between zonulin and inflammation and between zonulin and IP still needs to be determined.

## 4. Dysbiosis as a Marker of Intestinal Barrier Loss

Direct observation of intestinal barrier function implies practical difficulties; therefore, inspection mostly needs to be made indirectly. The microbiota is considered a barrier element of the host, which closely interacts with intestinal epithelial cells and with the immune and nervous systems, forming the bacteria-gut-brain axis [109].

The disruption of the finely tuned balance between microbiota and host is termed dysbiosis, and it can contribute to the loss of epithelial integrity, to the increase in IP, and to the weakening of defence mechanisms [110, 111]. Following increased IP induced by dysbiosis, bacterial products and metabolites (such as LPS, flagellin, bacterial DNA, and peptidoglycans) can permeate the epithelial barrier, thus reaching the liver and triggering an inflammatory response [112].

### 4.1. Indican and Skatole

Tryptophan is an essential amino acid for humans. Since it is not synthesised in the human body, commensal bacteria catabolise tryptophan in several different derivatives, which are absorbed by the intestine, enter the human metabolism, and are eliminated in the urine. Two of these compounds, indoxyl sulphate (otherwise known as indican) and 3-methyl-indole (skatole), are currently used to diagnose small intestinal dysbiosis and colon dysbiosis, respectively [113, 114].

For urinary indican determination, a standard colorimetric assay kit is used, while 3-methyl-indole assessment is performed by a high-performance liquid chromatography technique [115].

High concentrations of these compounds may reflect changes in bacterial growth in the small intestine, as well as malabsorption and constipation. It has been widely reported that these metabolites can be used as markers of altered gut microbiota in some extraintestinal diseases [116, 117]. Though small intestinal bowel bacterial overgrowth and undigested food components have been associated with the IBS-D subtype and have been implicated in the generation of symptoms [14, 118], there are no data about urinary levels of indican and skatole in this condition. Clinical studies that use these noninvasive dysbiosis parameters to study the effect of nutritional treatments in IBS-D patients on intestinal bacterial flora and the relationship with IP should be encouraged.

### 4.2. D-lactate

D-lactate is a metabolic end-product of GI bacteria. Since mammals do not have an enzyme system capable of decomposing D-lactate, D-lactate enters the blood when intestinal barrier function is damaged. Typically, serum levels of D-lactate in mammals are relatively low. Nevertheless, lesions of the intestinal epithelium and increased IP can cause an outflow of bacteria and products of their metabolism, including D-lactate, into the circulation [119]. Previous studies in animal models and humans have reported that plasma D-lactate levels increase soon after damage to the intestinal mucosal barrier [120, 121]. Thus, D-lactate accumulation in the systemic circulation can generally be considered a result of bacterial overgrowth and an increase in gut permeability induced by some GI disorders [22, 122, 123].

In a paper by Song et al. [124], it was observed that serum D-lactate was a potential marker of intestinal ischaemia severity and could be utilised as an important monitoring index in the treatment of IBD. Regarding IBS-D, recent studies have shown that serum D-lactate and DAO levels are significantly higher in this intestinal disease than in controls. In addition, good correlation with other barrier markers (such as urinary La/Ma ratio and mucosal expression of TJ-related proteins) has been observed, confirming the existence of gut barrier dysfunction [40, 125]. These preliminary results support the hypothesis describing significant involvement of dysbiosis and IP in the pathophysiology of IBS-D. Therefore, the peripheral blood levels of this metabolite could serve as an indicator of impaired barrier function in association with the La/Ma test. Nevertheless, given the limited clinical use, serum D-lactate levels as markers of an altered intestinal barrier should be confirmed with additional research.

## 5. Conclusion and Future Perspectives

Structural and functional alterations of the intestinal epithelial barrier challenge the classical conception of IBS as a purely functional disorder and provide evidence of an organic basis of the disease. Moreover, these modifications suggest that gut barrier dysfunction represents a crucial pathophysiological mechanism, particularly in IBS-D patients. From a therapeutic point of view, noninvasive monitoring of intestinal barrier function has become relevant. The use of noninvasive biomarkers of barrier integrity is accepted by the patients. In addition, many biomarkers can easily be measured using a simple technique such as ELISA. Regarding the functional assessment of IP, most of the available tests are time-consuming, and several confounding factors can hamper the interpretation of the results. Moreover, there are no standardised protocols (probes, cut-off values used, test duration, and fasting times), resulting in differences across studies, which make comparisons difficult [50, 117]. Each test that evaluates intestinal integrity and permeability also has its specific advantages and disadvantages and requires a particular detection method [13].

Therefore, performing a combination of methods to obtain information on the different aspects of the intestinal barrier has been suggested, particularly when functional and imaging techniques are combined with biomarker analysis. This approach has not yet been tested and can be essential for future research. In this context, the combination of a permeability test using sugars as probes (i.e., La and Ma) with intestinal epithelial integrity tests, such as plasma I-FABP and DAO, could be recommended, particularly in IBS-D patients. Following this approach, it has been observed that intestinal barrier injury and low-grade inflammation are present in a subgroup of IBS-D patients [29]. Consequently, intervening with personalised, targeted therapy could be possible. Moreover, persistent altered barrier function could predispose individuals to IBS; thus, the availability of simple, reliable, and sensitive methods for measuring IP may help to identify populations at risk of developing this condition as well as other GI diseases based on alterations of barrier functions (e.g., IBD, NCWS, and CD) and may aid in their therapeutic management.

## Figures and Tables

**Table 1:** Noninvasive methods for assessment of intestinal barrier integrity and function.

Probe	Test site	Method	Indicative for	Samples	Advantages	Limitations
*I-FABP*	Small intestine	ELISA	Epithelial integrity	Blood/urine	Region-specific	Acute phase
*TJ proteins*	Whole intestine	Western blot analysis	Epithelial integrity	Urine	Detection of TJ loss without tissue sections	Nonspecific for gut
*DAO*	Small intestine	ELISA/enzymatic spectrophotometry	Epithelial integrity	Blood	Marker of maturation and integrity	Limited data
*^51^Cr-EDTA*	Whole intestine	Gamma counter	Epithelial function	Urine	Easy detection, not naturally present	Radioactivity, single probe
*PEG*	Whole intestine	HPLC, LC-MS	Epithelial function	Urine	Not metabolized by enzymes or degraded by bacteria within the human GI tract	Laborious detection
*La/Ma*	Small intestine	HPAEC-PAD/LC-MS	Epithelial function	Urine	Combination in multisugar tests, widely used	Time-consuming
*Sucrose*	Stomach-duodenum	HPAEC-PAD/LC-MS	Epithelial function	Urine	Specific for stomach	Time-consuming, degraded by sucrase in the duodenum
*Sucralose*	Whole intestine	HPAEC-PAD/LC-MS	Epithelial function	Urine	Resistive to bacterial degradation	Time-consuming, long collection time
*Zonulin*	Whole intestine	ELISA	Epithelial function	Blood/feces	Specific for the small intestine, correlation with IP	Low ELISA specificity for detection
*D-lactate*	Whole intestine	ELISA/enzymatic spectrophotometry	Epithelial integrity	Blood	Easy detection	Limited data in humans

I-FABP: intestinal fatty acid-binding protein; TJs: tight junctions; DAO: diamine oxidase; ^51^Cr-EDTA: 51Chromium-labeled Ethylene Diamine Tetra-acetic Acid; PEG: polyethylene glycols; La/Ma: lactulose to mannitol; ELISA: enzyme-linked immunosorbent assay; HPLC: high-performance liquid chromatography; LC-MS: liquid chromatography-mass spectrometry; HPAEC-PAD/LC-MS: high-performance anion-exchange chromatography with pulsed amperometric detection.

**Table 2:** Intestinal permeability (IP) measured with the La/Ma ratio identifies two subtypes of IBS-D patients.

Author	Year	IBS-D patients/controls	IP of IBS-D patients (% above normal)	Cut-off La/Ma	Comments	Ref
*Linsalata*	2018	39/20	46%	0.035	Increased levels of I-FABP, DAO, IL-6, and LPS	29
*Zhou*	2009	54/22	39%	0.07	Higher M-VAS pain intensity reading scale and higher FBDSI score	70
*Zhou*	2010	19/10	42%	0.07	Decreased glutamine synthetase expression, increased miR-29a expression	71
*Li*	2016	40/10	47%	0.025	Higher claudin-4 expression, 185 genes differentially expressed, worse psychological effects, and quality of life	72
*Russo*	2018	34/17	44%	0.035	Higher IL-6, lower GSRS cluster pain, and diarrhea profile	73

IBS-D: irritable bowel syndrome with diarrhea; IP: intestinal permeability; La/Ma: lactulose to mannitol; I-FABP: intestinal fatty acid-binding protein; DAO: diamine oxidase; IL-6: interleukin-6; LPS: lipopolysaccharides; FBD: Functional Bowel Disorder; VAS: Visual Analogue Score; GSRS: Gastrointestinal Symptom Rating Score.

## Data Availability

The data supporting this literature review are from previously reported studies that have been cited. No original data have autonomously been processed by the authors during the preparation of the manuscript.

## Abbreviations

CD: Celiac disease
^51^Cr-EDTA: 51-Chromium-labeled Ethylene Diamine Tetra-acetic Acid
DAO: Diamine oxidase
E: Erythritol
ELISA: Enzyme-linked immunosorbent assay
FBD: Functional Bowel Disorder
GFD: Gluten-Free Diet
GI: Gastrointestinal
GSRS: Gastrointestinal Symptom Rating Score
HC: Healthy controls
HPAEC-PAD: High-performance anion-exchange chromatography with pulsed amperometric detection
IBD: Inflammatory bowel disease
IBS: Irritable bowel syndrome
IBS-D: Irritable bowel syndrome with diarrhea
IBS-C: Irritable bowel syndrome with constipation
IBS-M: Mixed irritable bowel syndrome
I-FABP: Intestinal fatty acid-binding protein
IL: Interleukin
IP: Intestinal permeability
La: Lactulose
LC-MS: Liquid chromatography-mass spectrometry
LPS: Lipopolysaccharides
Ma: Mannitol
miRNAs: MicroRNAs
NCWS: Nonceliac wheat sensitivity
NPI-IBS: Non-postinfectious IBS
PEG: Polyethylene glycols
PI-IBS: Postinfectious IBS
R: Rhamnose
S: Sucralose
Su: Sucrose
TJs: Tight junctions
VAS: Visual Analogue Score.
